# Inflorescence Meristem Fate Is Dependent on Seed Development and FRUITFULL in *Arabidopsis thaliana*

**DOI:** 10.3389/fpls.2019.01622

**Published:** 2019-12-18

**Authors:** Vicente Balanzà, Irene Martínez-Fernández, Shusei Sato, Martin F. Yanofsky, Cristina Ferrándiz

**Affiliations:** ^1^Instituto de Biología Molecular y Celular de Plantas, Consejo Superior de Investigaciones Científicas, Universidad Politécnica de Valencia, Valencia, Spain; ^2^Division of Biological Sciences, University of California San Diego, San Diego, CA, United States

**Keywords:** inflorescence meristem, terminal flower, inflorescence development, inflorescence proliferative arrest, FRUITFULL, AGAMOUS

## Abstract

After a vegetative phase, plants initiate the floral transition in response to both environmental and endogenous cues to optimize reproductive success. During this process, the vegetative shoot apical meristem (SAM), which was producing leaves and branches, becomes an inflorescence SAM and starts producing flowers. Inflorescences can be classified in two main categories, depending on the fate of the inflorescence meristem: determinate or indeterminate. In determinate inflorescences, the SAM differentiates directly, or after the production of a certain number of flowers, into a flower, while in indeterminate inflorescences the SAM remains indeterminate and produces continuously new flowers. Even though indeterminate inflorescences have an undifferentiated SAM, the number of flowers produced by a plant is not indefinite and is characteristic of each species, indicating that it is under genetic control. In *Arabidopsis thaliana* and other species with indeterminate inflorescences, the end of flower production occurs by a regulated proliferative arrest of inflorescence meristems on all reproductive branches that is reminiscent of a state of induced dormancy and does not involve the determination of the SAM. This process is controlled genetically by the FRUITFULL-APETALA2 (FUL-AP2) pathway and by a correlative control exerted by the seeds through a mechanism not well understood yet. In the absence of seeds, meristem proliferative arrest does not occur, and the SAM remains actively producing flowers until it becomes determinate, differentiating into a terminal floral structure. Here we show that the indeterminate growth habit of *Arabidopsis* inflorescences is a facultative condition imposed by the meristematic arrest directed by FUL and the correlative signal of seeds. The terminal differentiation of the SAM when seed production is absent correlates with the induction of *AGAMOUS* expression in the SAM. Moreover, terminal flower formation is strictly dependent on the activity of FUL, as it was never observed in *ful* mutants, regardless of the fertility of the plant or the presence/absence of the *AG* repression exerted by APETALA2 related factors.

## Introduction

For most plants, reproductive success depends on the ability to produce seeds that ensure the perpetuation of the species. Seed production is related to the number of flowers produced by the plant during the reproductive phase, and therefore, dependent on the activity of the inflorescence meristems that produce the flowers. Inflorescences have been classified in two major categories based on the fate of the inflorescence meristem: determinate or indeterminate. Determinate inflorescences are those where the shoot apical meristem (SAM) differentiates directly, or after the production of a certain number of flowers, into a flower. Thus, the end of the reproductive phase in determinate inflorescences is established by the final differentiation of the inflorescence SAM, determining the final number of flowers and seeds produced per shoot. On the other hand, indeterminate inflorescences are those where the SAM remains undifferentiated and produces continuously new flowers until the end of the reproductive phase. Interestingly, the length of the reproductive phase and the number of flowers produced by indeterminate inflorescences is finite, despite the undifferentiated nature of the SAM, which does not produce any terminal structure. Moreover, in indeterminate inflorescences, the number of flowers produced before SAM arrest is usually distinctive for each species and/or ecotype, suggesting a genetic control of the length of the reproductive phase.

Despite of the developmental differences between them, determinate and indeterminate inflorescences can be found in multiple plant families, as well as in plants with different growth habits, like in monocarpic (reproducing only once during their life cycle) or polycarpic plants (alternating reproductive and vegetative phases). In monocarpic plants with indeterminate inflorescences, the end of the reproductive phase has been associated with a process named Global Proliferative Arrest (GPA). GPA has been well characterized for the model species *Arabidopsis thaliana* (Hensel et al., 1994). During GPA, after the production of a determined number of flowers, the SAM arrests its growth, and all floral buds, up to the last non-pollinated flowers, do not develop further. In a short period of time, all active meristems in the plant undergo the same process. At this point, fruit filling and seed maturation is completed in all fertilized flowers and then, the plant senesces and dies. Although the end of the flowering phase might be assumed as a default process, linked to meristem exhaustion and plant senescence, classical studies indicate that it is a regulated process, preceding senescence of reproductive branches in polycarpic species or of the whole plant in annual species (Murneek 1926; Leopold et al., 1959; Lindoo and Nooden, 1977; Hensel et al., 1994; Wilson, 1997; Noodén et al., 2004). It has been proposed that proliferative arrest could be related with the proper allocation of nutrients to the developing seeds, and, thus, the establishment of strong source-sink relationships between the seeds and the inflorescence meristem could restrict plant growth and trigger the end of flowering (Sinclair and de Wit, 1975; Kelly and Davies, 1988). In agreement with this, the major factor controlling the end of flowering is seed production, as proven by the extended flowering period of plants with strongly reduced fertility (Murneek, 1926; Leopold et al., 1959; Lindoo and Nooden, 1977; Hensel et al., 1994; Wilson 1997; Noodén et al., 2004). The mechanism of this correlative control exerted by the seeds is still unknown (Walker and Bennett, 2018), but it has been shown that it modifies the SAM activity, inducing a state reminiscent of meristem dormancy, with low mitotic activity, a reduction of reactive oxygen species, and accumulation of abscisic acid response genes (Wuest et al., 2016).

In addition to the correlative control of seeds, the end of the reproductive phase in *Arabidopsis* indeterminate inflorescences is also controlled genetically by a recently described pathway likely dependent on the age of the inflorescence (Balanza et al., 2018). Briefly, APETALA2 (AP2) and other related factors of the same family sustain the expression of *WUSCHEL* (*WUS*), a key gene involved in stem cell maintenance, in the inflorescence meristem (Laux et al., 1996; Mayer et al., 1998). FRUITFULL (FUL), a MADS-box transcription factor involved in multiple developmental processes and strongly expressed in the inflorescence meristem, directly represses the expression of *AP2* and *AP2*-*like* genes in this domain (Gu et al., 1998; Ferrandiz et al., 2000a; Ferrandiz et al., 2000b; Shikata et al., 2009; Wang et al., 2009; Yamaguchi et al., 2009; Balanza et al., 2014; Bemer et al., 2017). The genes in the *AP2* clade are also negatively regulated by the action of the miR172 in an age dependent way (Aukerman and Sakai, 2003; Chen, 2004; Wang et al., 2009; Wu et al., 2009). Based on the phenotypes of the different mutants, we previously proposed that the combined action of miR172 and FUL, increasingly accumulated through inflorescence development, would lead to decreasing levels of AP2 and AP2-like factors in the SAM, eventually unable to maintain WUS activity. Accordingly, *ful* mutants and *AP2* alleles resistant to the action of miR172 delay the end of the flowering phase, resulting in an increased flower production (Balanza et al., 2018).

Interestingly, in *Arabidopsis* sterile mutants, or in wild type plants where flowers are removed, the end of the reproductive phase differs from that observed in fertile plants. As mentioned above, sterile mutants produce more flowers than fertile plants, and instead of ending flower production with meristem arrest, the inflorescence meristem of sterile mutants become determinate producing a terminal flower of carpelar nature (Chaudhury 1993; Schultz and Haughn, 1993; Hensel et al., 1994; Modrusan et al., 1994). The nature of this shift in the inflorescence SAM fate is still unknown, although it could be interpreted as a differentiation of the SAM into a floral meristem, similar to that observed in determinate inflorescences. The major factors that control which meristems become flowers and which remain as undifferentiated shoots have been studied in model species but also in many other plants. In the *Arabidopsis* indeterminate inflorescence, once floral transition has taken place, the lateral meristems produced by the SAM acquire floral identity. The main factors that control the specification of floral fate in these meristems are LEAFY (LFY) and APETALA1 (AP1), which confer floral meristem identity upstream of floral organ identity factors (Mandel et al., 1992; Weigel et al., 1992; Weigel and Meyerowitz, 1993). The SAM remains indeterminate by restricting the expression of these floral promoting factors, which are excluded by the activity in this domain of TERMINAL FLOWER1 (Shannon and Meeks-Wagner, 1991; Liljegren et al., 1999; Ratcliffe et al., 1999), homologous to phosphatidylethanolamine binding proteins and a member of a small gene family, which includes *FLOWERING LOCUS T (FT)*, a key regulator of flowering time regarded as the florigen (Wigge et al., 2005). The antagonistic interaction between TFL1 and AP1/LFY is crucial to maintain the indeterminacy of the *Arabidopsis* inflorescence. In *Arabidopsis tfl1* loss of function mutants, the inflorescence meristem ectopically expresses *AP1* and *LFY* differentiating prematurely into a flower, and thus, the inflorescence becomes determinate (Shannon and Meeks-Wagner, 1991; Liljegren et al., 1999; Ratcliffe et al., 1999). Likewise, plants overexpressing *AP1* or *LFY*, and even some of their downstream floral organ identity factors, like *AGAMOUS* (*AG*), also cause the conversion of the SAM into a flower (Mizukami and Ma, 1992; Mandel and Yanofsky, 1995b; Blazquez et al., 1997). The basic TFL/AP1/LFY module has been characterized in many other species and shown to be largely conserved in angiosperms, and variations in its configuration appear to correlate well with determinate/indeterminate growth habits (Pnueli et al., 1998; Liljegren et al., 1999; Ratcliffe et al., 1999; Benlloch et al., 2007; Mimida et al., 2011; Mohamed et al., 2010; Imamura et al., 2011; Iwata et al., 2012).

Here, we show that the FUL-AP2 pathway that regulates the length of the flowering phase in fertile plants is also controlling the fate of the inflorescence meristem in sterile plants. We show that the formation of the terminal flower in the absence of seeds associates with the ectopic expression in the SAM of the *AG* gene. Loss of AP2 function leads to sterile plants that produce an early differentiation of the inflorescence meristem into a terminal flower, suggesting that AP2 participates in the repression of *AG* in the SAM. Finally, we also show that the ectopic activation of *AG* in the SAM is strictly FUL-dependent, as *ful* mutants suppress the terminal flower formation in the absence of seeds, even in 35S:miR172 background where AP2-like factors are not active.

## Results

### Meristem Fate Is Dependent on the Presence of Seeds and FUL Activity

It has been described that seed development directly impacts the end of the flowering phase in *Arabidopsis thaliana* inducing the SAM arrest. In the absence of seeds, the SAM remains active for longer and the number of flowers produced is increased. In these conditions, the SAM will produce new flowers until it differentiates into a terminal floral structure, resembling the behavior of a determinate inflorescence, and also of mutants where inflorescence meristem identity is compromised (Hensel et al., 1994; Ohshima et al., 1997; Bradley et al., 1997). As the FUL-AP2 pathway genetically controls the length of the flowering phase, we decided to assess if the FUL-AP2 module had also a role in the determination of the fate of the inflorescence meristem and the formation of the terminal structure.

**Figure 1:**
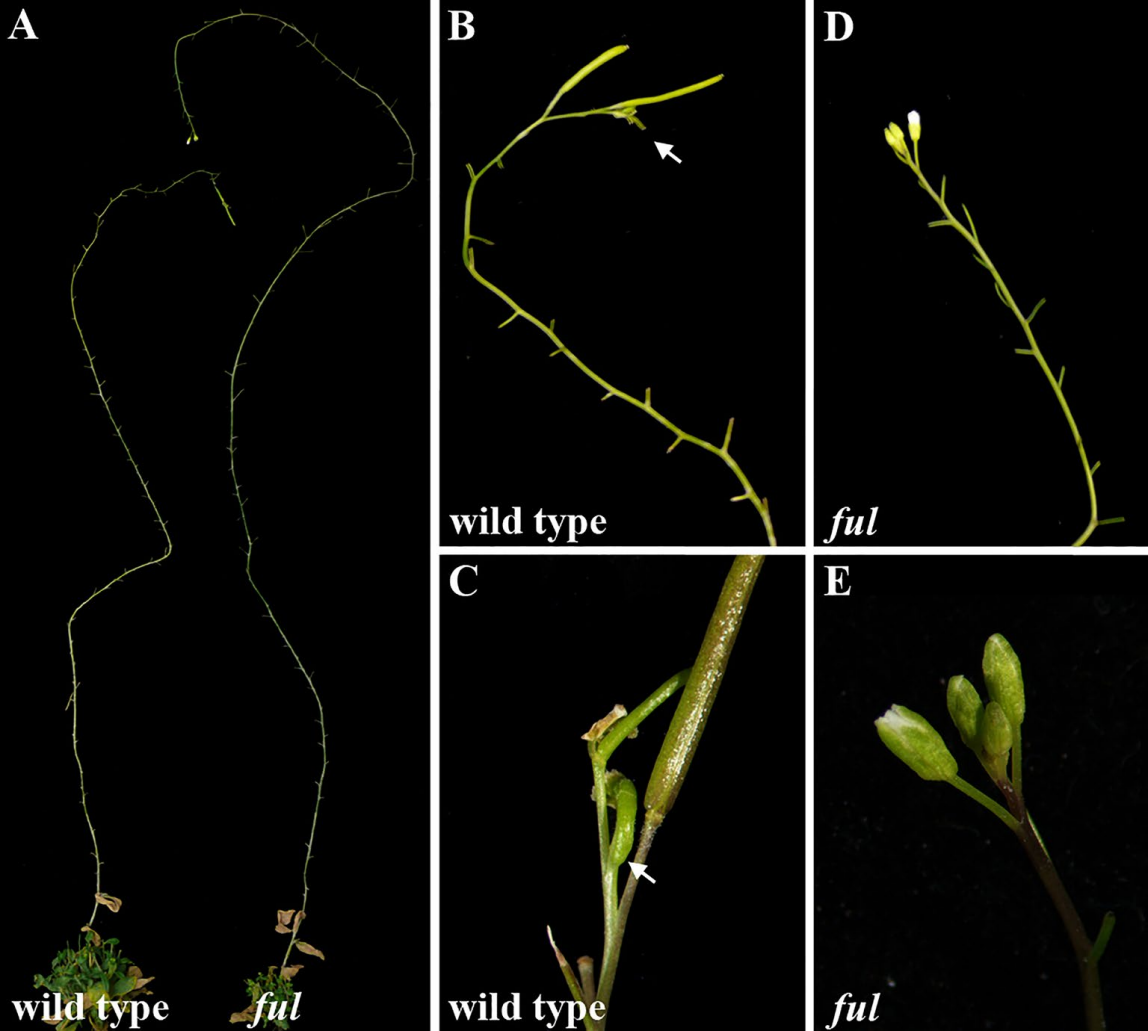
Inflorescence phenotype of pruned 14-week-old plants. All branches, as well as flowers in anthesis stage were removed in wild type and *ful* mutant plants **(A)**. Plants stay alive for longer, delaying the end of flowering. At the end of the flowering phase, wild type inflorescence becomes determinate **(B**, **C)**, producing a terminal flower (arrow) while *ful* mutant remains indeterminate **(D**, **E)**.

For this purpose, *ful* mutants were grown in the absence of seed production, by continuously removing all the flowers produced by the SAM at anthesis. As described previously, wild type plants and *ful* mutants responded to the pruning by increasing the number of flowers produced by the SAM (Balanza et al., 2018) (**Figure 1A**). Surprisingly, while the wild type SAM eventually differentiated into a terminal flower (**Figures 1B, C**), the SAM of *ful* mutants remained undifferentiated, generating flowers until the death of the plant (**Figures 1D, E**), without producing the terminal structure observed in the wild type.

**Figure 2:**
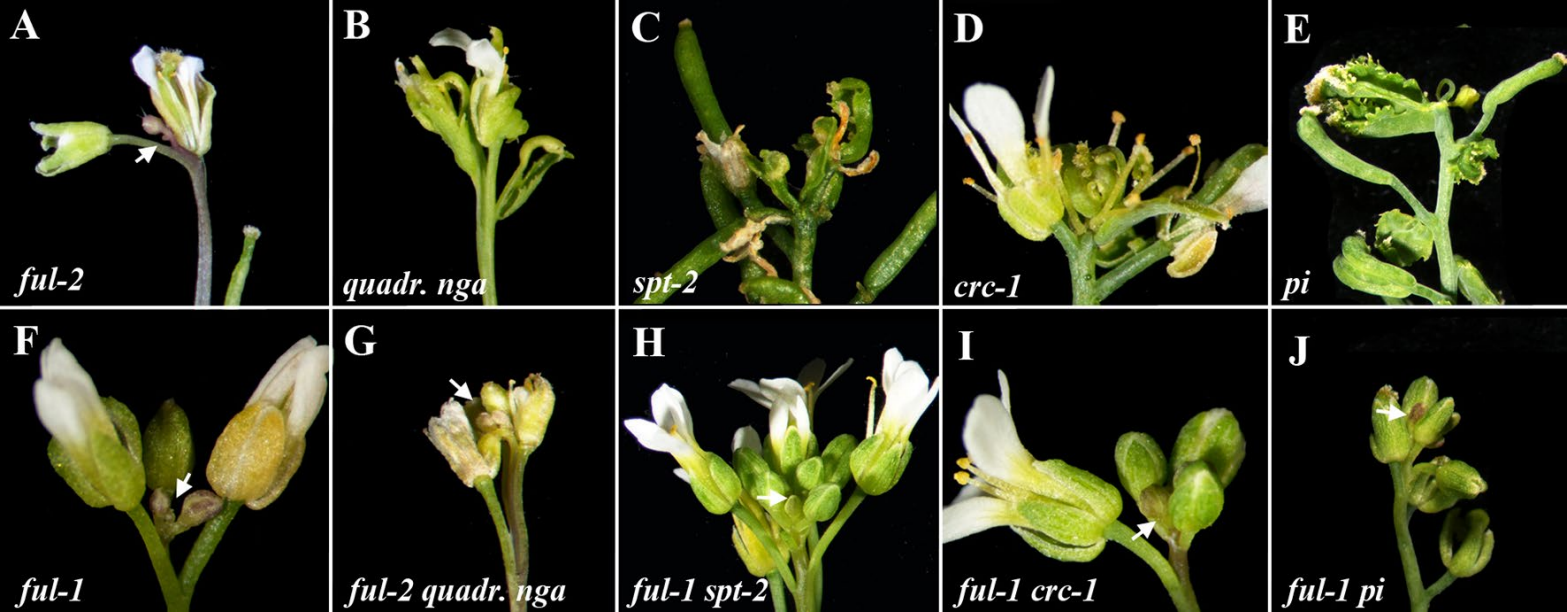
Terminal flower formation is never observed in *ful* mutants. *ful* mutants show an extended flowering phase with a morphologically distinct inflorescence meristem always present that remains active **(A**, **F)**. Mutants with strongly reduced fertility show inflorescences that terminate with the formation of a terminal flower as shown for the quadruple *nga* **(B)**, *spt-2* **(C)**, *crc-1* **(D)**, or *pi* **(E)**. When these sterile mutants are combined with *ful*, the formation of the terminal structure is suppressed and meristems remain active until the death of the plant **(G**, **H**, **I**, **J)**.

To confirm that the observed phenotype in manually pruned *ful* plants was independent of the treatment, we decided to characterize inflorescence meristem fate in mutant combinations of *ful* with unrelated mutations previously described that caused sterility or a severe reduction of fertility, such as *crabs claw* (*crc*) (Bowman and Smyth, 1999), *spatula* (*spt*) (Alvarez and Smyth, 1999), the quadruple *ngatha* (*nga*) mutant (Trigueros et al., 2009), or *pistillata* (*pi*) (Hill and Lord, 1989). While all the sterile mutants tested ended flowering with the formation of the typical terminal flower (**Figures 2B–E**), in combination with *ful* they remained active for longer without SAM differentiation, similarly to what was observed in the *ful* pruning experiment, and ending flowering as the *ful* single mutant (**Figures 2A, F–J**). These phenotypes suggested a role for FUL in controlling inflorescence meristem fate, where it could act by inducing the formation of the terminal structure observed in plants that did not produce seeds.

### *AG* Is Expressed Ectopically in the Inflorescence Shoot Apical Meristem Under Sterile Conditions

**Figure 3:**
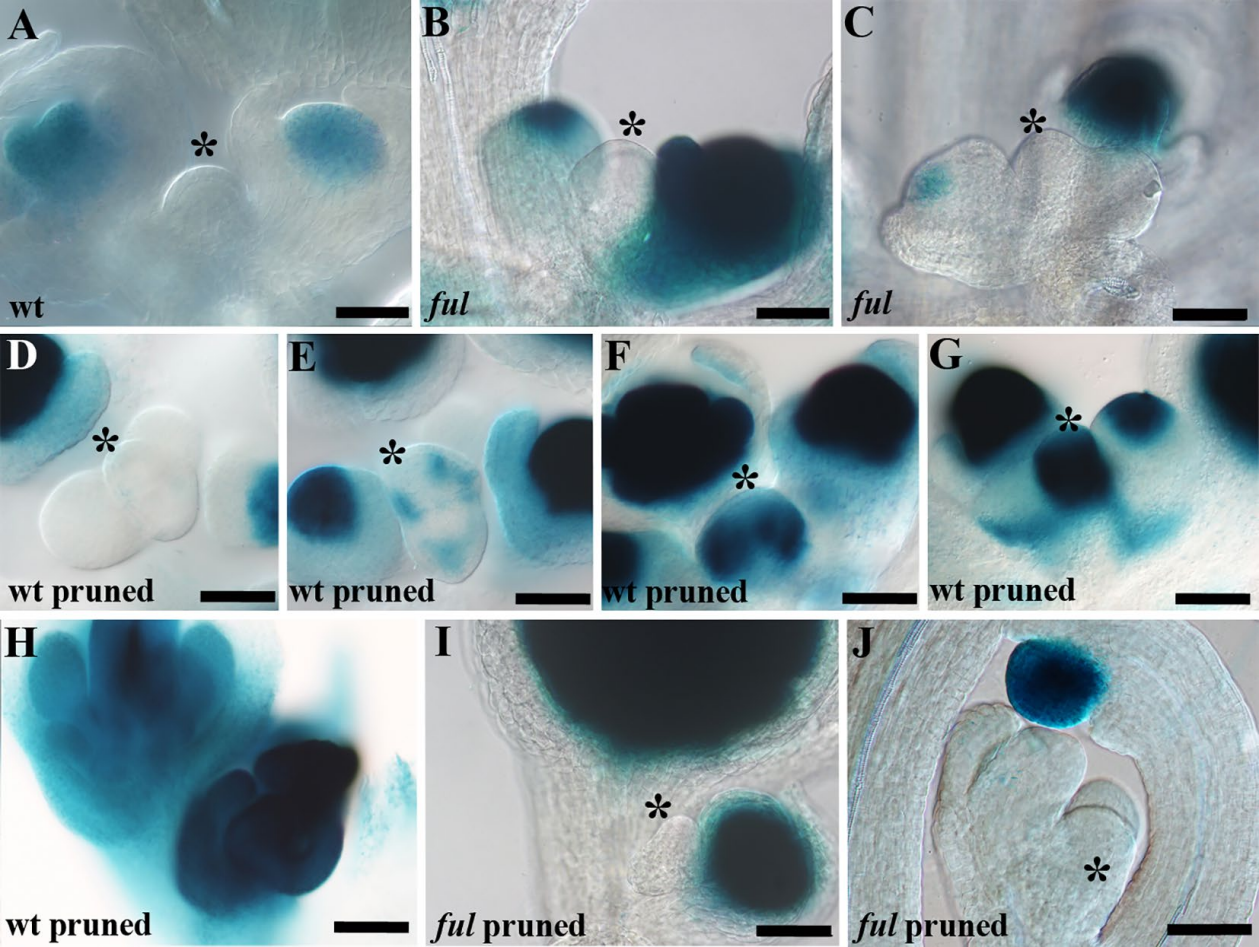
*AGAMOUS* expression at the end of the flowering phase. **(A)** Inflorescence shoot apical meristem (SAM) of a fertile wild type plant in Global Proliferative Arrest, at the end of the flowering phase, around 3–4 weeks after floral transition. AG::GUS reporter activity is detected in the central whorls of the flowers, but never in the SAM. **(B)** *ful* mutant inflorescence SAM, at 4 weeks after floral transition. As for wild type plants of same age, no AG::GUS activity is observed in the SAM **(C)** *ful* mutant inflorescence SAM, at 6 weeks after floral transition. Even 2–3 weeks after the arrest of the wild type plants, no AG::GUS activity is detected in the SAM. **(D)** Wild type plants where sterility was induced by pruning of flowers, 5 weeks after floral transition, still proliferative. AG::GUS signal is identical to that observed in control plants during the proliferative phase of the inflorescence. **(E)** Inflorescence meristem of pruned wild type plants at 5–6 weeks after floral transition. Preceding the visible morphological differentiation of the terminal structure, the GUS signal starts to be detected in the periphery of the SAM. **(F–G)** Inflorescence meristem of pruned wild type plants at 6–7 weeks after floral transition. AG::GUS signal extends to the whole SAM. **(H)** Terminal structure of a pruned wild type plant, 7 weeks after floral transition, showing high AG::GUS activity. **(I)** Pruned *ful* mutant, 7 weeks after floral transition. **(J)** Pruned *ful* mutant, 9 weeks after floral transition. In pruned ful mutants which never form a terminal flower, the GUS signal was never detected in the SAM. Black bars represent 50 μm. Asterisk indicates the SAM.

AG is a homeotic MADS-domain transcription factor that confers carpel identity during flower development (Yanofsky et al., 1990; Bowman et al., 1991). It has been described that the constitutive expression of *AG* induces the differentiation of the inflorescence meristem into a terminal structure after the production of a reduced number of flowering nodes (Mizukami and Ma, 1992). As the terminal structure observed in sterile plants was mainly composed by carpel-like structures, we decided to check *AG* expression throughout inflorescence development in the presence (untreated wild type plants) or absence of seed development (pruned plants) by monitoring the activity of an AG::GUS reporter previously generated and characterized (Sieburth and Meyerowitz, 1997). As expected, in untreated wild type plants no β-glucuronidase (GUS) signal was observed in the SAM at any inflorescence developmental stage where the meristem was proliferative and producing new flowers (**Supplementary Figure 1**), while the signal was clearly detected in the center of the floral meristems. The lack of AG::GUS expression in the SAM was also evident in the arrested inflorescence meristems at the end of the flowering period 3–4 weeks after bolting, when no further initiation or development of floral buds was taking place (**Figure 3A**). In pruned plants, where seed production was avoided, the GUS signal pattern was identical to that of the fertile control plants during the proliferative phase of inflorescence development (**Supplementary Figure 1**), being only present in the center of floral buds but absent in the SAM. However, the AG::GUS reporter activity was clearly detected in the SAM of pruned plants at late stages of inflorescence development, preceding the formation of the terminal flower. In pruned plants, AG::GUS signal started to be detected in the periphery of the SAM at 6–7 weeks after bolting (**Figures 3D, E**) to later extend to the entire SAM (**Figures 3F, G**) until its differentiation in the terminal structure (**Figure 3H**). Our results indicate that the terminal carpelar structure observed at the end of the flowering phase in the absence of seed production is associated with the ectopic expression of *AG* in the SAM.

As the *ful* mutant does not undergo SAM differentiation into a terminal structure in the absence of seed production, we decided to analyze the activity of the AG::GUS reporter in *ful* plants where flowers were continuously removed. In agreement with the phenotypes observed when this treatment was applied in *ful* mutants, the AG::GUS signal was only detected in the floral meristems in both *ful* AG::GUS untreated (fertile) (**Figures 3B, C**) and pruned (sterile) plants (**Figures 3I, J**), but never in the SAM, even 2 or 3 weeks after the formation of the terminal flowers in the pruned wild type control plants (**Figures 3I, J**) (8–9 weeks after bolting). These results strongly suggested that the activation of *AG* in the SAM in the absence of seed development was FUL-dependent.

### *AP2-Like* Genes Repress *AG* in the Shoot Apical Meristem

**Figure 4:**
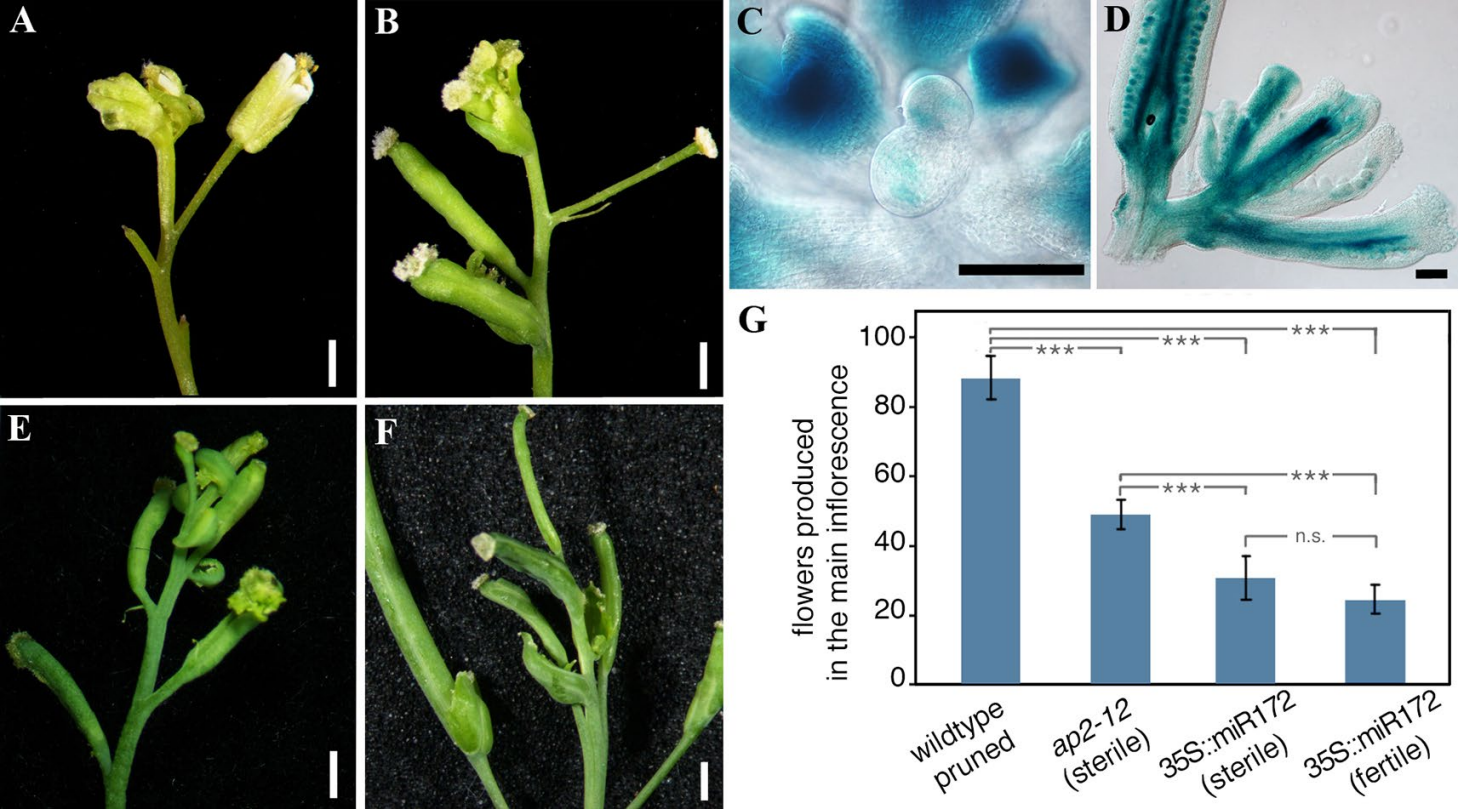
AP2 prevents terminal flower formation. **(A)** Pruned/wild type inflorescence 6 weeks after the floral transition. The inflorescence meristem differentiates into a terminal carpeloid structure. **(B)** The sterile *ap2–12* mutant also ends flowering with the formation of an early terminal flower 3–4 weeks after floral transition. **(C)** The terminal floral structure observed in the *ap2–12* mutant is preceded by ectopic AG expression in the SAM 3 weeks after floral transition. **(D)** AG::GUS activity is strongly detected in the terminal structure of *ap2–12* inflorescences 4 weeks after floral transition. **(E)** The sterile 35S::miR172 plants also end the flowering phase with the formation of the terminal floral structure around 2 weeks after bolting. **(F)** A similar terminal flower formation is also observed in the 35S::miR172 that occasionally developed fertile pods, suggesting that the formation of the terminal floral structure depends on the activity level of AP2-like genes. **(G)** Flower production before terminal flower formation in the main inflorescence of wild type pruned plants, *ap2–12* mutants (sterile), 35S::miR172 sterile lines, and 35S::miR172 fertile lines. The number of flowers produced in the lines where AP2 and AP2-like activity is reduced is much lower than in wild type plants where seed production was avoided. Error bars represent s.d. A pair-wise Student’s t-test, correcting with Holm method for multiple testing and linked to a *post-hoc* analysis, was performed to indicate genotypes with significant differences. *** indicate a significant difference (P < 0.001), n.s., not significative. N≥ 15. Black bars in **(C**, **D)** represent 100 μm. White bars in **(A**, **B**, **E**, **F)** represent 1 mm.

In the floral meristem, AP2 is a classical repressor of *AG* acting in the two outer whorls of the flower (Bowman et al., 1991; Drews et al., 1991). In *ap2* loss-of-function mutants, *AG* becomes ectopically expressed in the external whorls of the flower producing the conversion of sepals to carpels and petals to staminoid structures. As FUL is a known repressor of *AP2* and other *AP2-like* genes in the SAM (Balanza et al., 2018), it could be expected that, in the *ful* mutants, increased *AP2* and *AP2-like* gene expression in the SAM could prevent *AG* upregulation in the inflorescence meristem at late stages in sterile plants. To test this hypothesis, we first characterized the end of the flowering phase of an *ap2* mutant compared to wild type. Since *ap2–12* is a sterile mutant, we compared it with a wild type control plant where seed production was avoided by pruning the developing flowers. *ap2–12* mutants produced far fewer flowers than the pruned control (Balanza et al., 2018), but both ended flowering with the formation of a terminal flower (**Figures 4A, B, G**), as expected for sterile backgrounds. Then, we wondered if the early determination of the inflorescence meristem observed in *ap2–12* could be related to an early activation of *AG* in the SAM. When we analyzed the AG::GUS reporter line in the *ap2* background we observed that, as in wild type pruned plants, the GUS signal was ectopically expressed in the SAM (**Figures 3D–H**, **Figures 4C, D**). Our results indicated that AP2 could mediate the repression of *AG* in this domain. To assess if the rest of the *AP2-like* genes also contributed to the repression of *AG* in the inflorescence meristem, we also characterized inflorescence development and inflorescence meristem fate in plants overexpressing miR172, where all genes in the *AP2* family are simultaneously down-regulated. All 35S::miR172 plants showed a characteristic early flowering phenotype, and most of them also showed the sepal-to-carpel homeotic conversions observed during flower development in the *ap2* mutant (Yant et al., 2010). Interestingly, a small fraction of the plants (around 20%) did not exhibit the homeotic transformations associated to the *ap2* floral phenotype, developing normal flowers that were fully fertile. As observed for the single *ap2–12* mutant, both phenotypic categories of 35S::miR172 plants had inflorescences that produced a terminal flower (**Figures 4E, F**), although inflorescence meristem determination occurred earlier than in the single *ap2–12* mutant, after the production of a further reduced number of flowers (Balanza et al., 2018) (**Figure 4G**). The earlier differentiation of the terminal flower in the 35S::miR172 plants, even in the presence of seeds (**Figure 4F**) indicates that not only *AP2*, but also the rest of *AP2-like* genes contribute to prevent the differentiation of the SAM into a terminal structure, possibly by jointly repressing *AG* in the SAM. Then, the increased levels of *AP2-like* gene expression in *ful* mutants (Balanza et al., 2018) could explain the suppression of the terminal flower differentiation in plants where seed production was prevented.

### FUL Is Required for the Ectopic Expression of *AG* in the Shoot Apical Meristem

**Figure 5:**
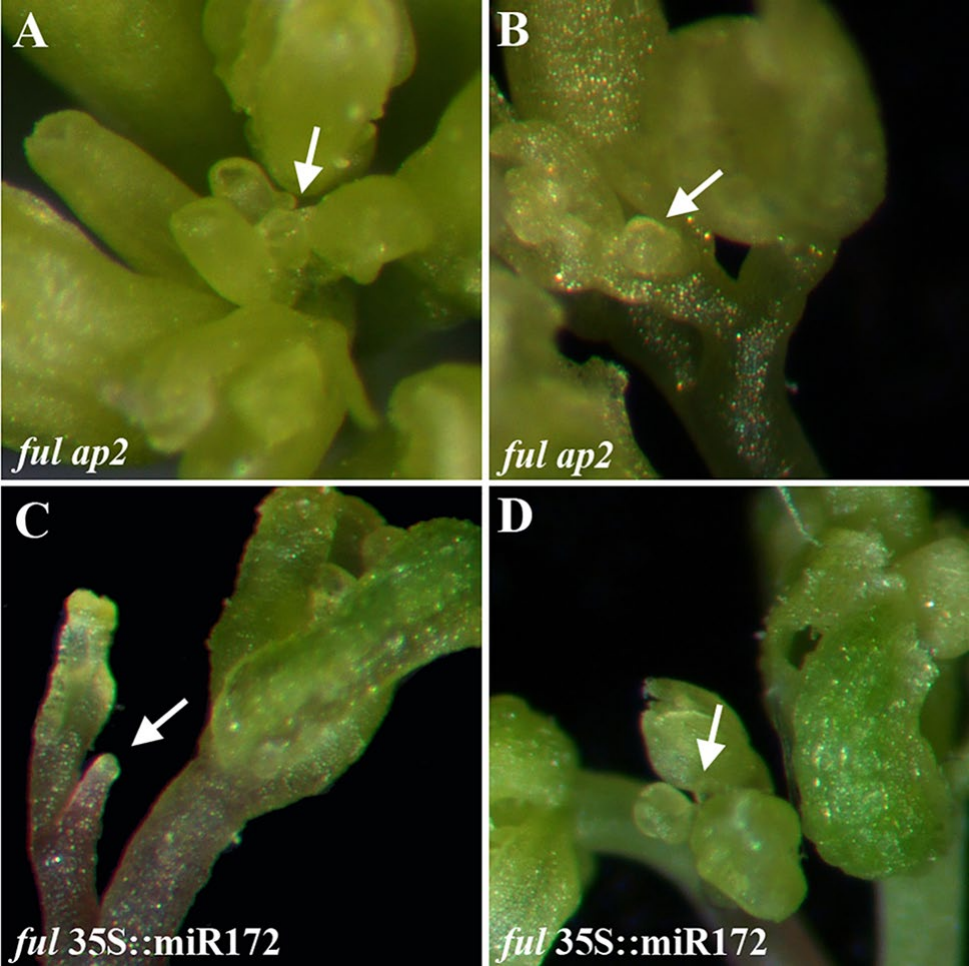
Terminal flower formation in the absence of AP2-like activity is dependent on FUL. The inflorescence meristem determination into a terminal structure observed in the *ap2–12* single mutant is suppressed by *ful* mutations **(A**, **B)**. In the 35S::miR172 plants, where the levels all the *AP2-like* genes are reduced and the terminal flower is formed very early in inflorescence development, the *ful* mutation also suppresses its formation **(C**, **D)**.

If the suppression of the terminal flower observed in *ful* mutants in the absence of seed production was mediated by the upregulation of *AP2* and *AP2-like* genes in the SAM, in a double *ful ap2* mutant we could expect the determination of the inflorescence meristem into a terminal structure. Surprisingly, the double *ful ap2* mutant, despite being sterile, ended the flowering phase without the formation of the terminal flower (**Figures 5A, B**). As in this double mutant the expression of other *AP2-like* genes still was elevated (Balanza et al, 2018), it was possible that this increased expression could be sufficient to suppress the formation of the terminal structure. To test this hypothesis, we checked inflorescence meristem fate in a *ful* 35S::miR172 line. In this genetic combination, the levels of all the AP2-like proteins are downregulated by the overexpression of miR172 even in the absence of FUL activity. As described previously, the *ful* 35S::miR172 plants produced a similar number of flowers to 35S::miR172 plants, suppressing the increased flowering period and delayed proliferative arrest observed in the single *ful* mutant, and therefore indicating that the FUL effect on the duration of the flowering phase is mediated by AP2-like factors (Balanza et al., 2018). Surprisingly, the differentiation of the inflorescence meristem into a terminal flower that occurred in 35S::miR172 plants was never observed in *ful* 35S::miR172 lines (**Figures 5C, D**). These results indicate that to promote the formation of the terminal floral structure and probably the ectopic *AG* expression in the SAM, it is necessary the downregulation of *AP2-like* genes and the presence of FUL in the inflorescence meristem at later stages of inflorescence development.

**Figure 6:**
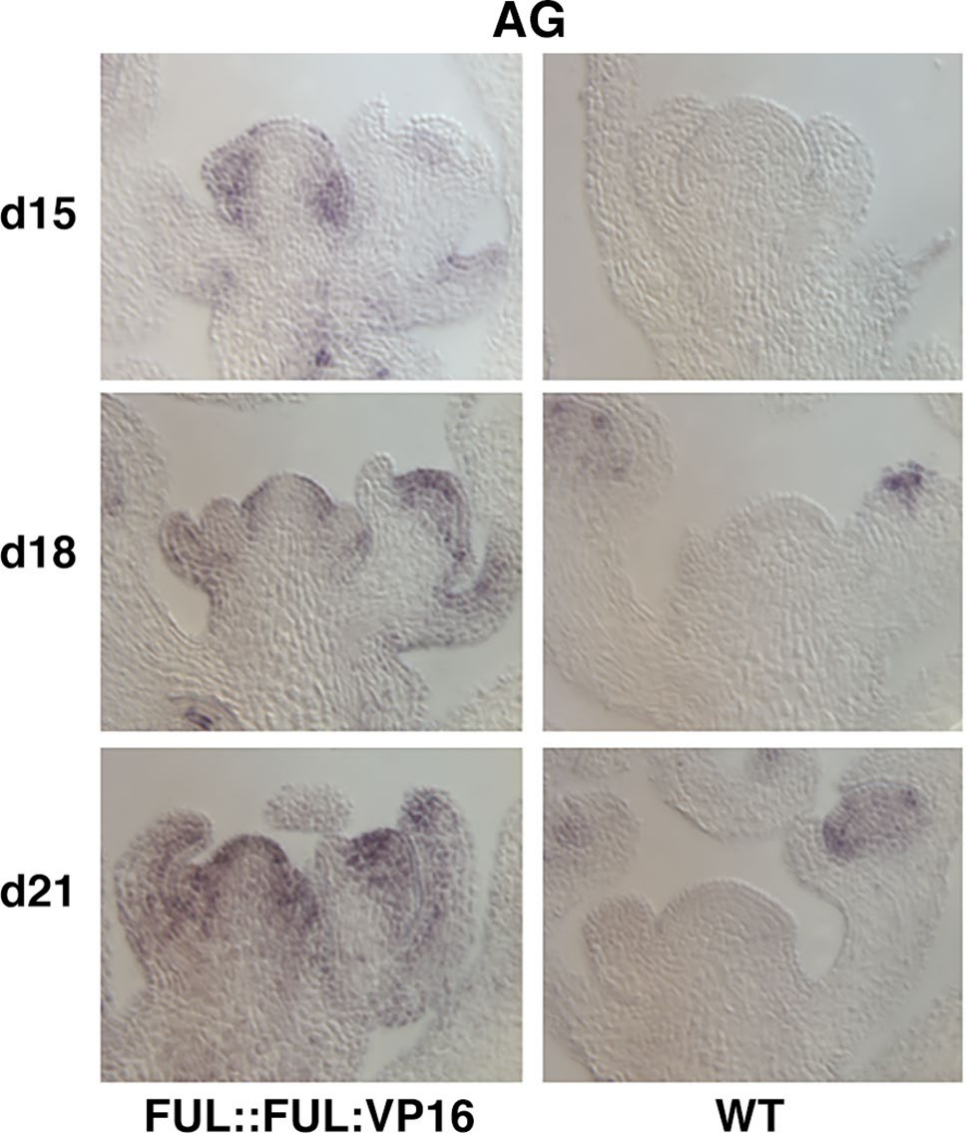
AG ISH on inflorescences of FUL::FUL:VP16 plants. *AG* expression was detected by ISH on FUL::FUL:VP16 (left) and wild-type (right) plants at early stages of inflorescence development. Samples were collected 15, 18, and 21 days after germination. While in the wild-type plants *AG* expression was only detected in the center of floral meristems, in FUL::FUL:VP16 plants *AG* expression was detected ectopically in the periphery of the shoot apical meristem (SAM) at 18 days after germination (approx. 1 week after floral transition), being present throughout the SAM 21 days after germination (approx. 2 weeks after floral transition).

The requirement of FUL to induce the differentiation of the inflorescence meristem into a terminal flower suggested that FUL could work as an *AG* activator. To confirm the ability of FUL to activate *AG*, we analyzed the expression of *AG* in a FUL::FUL:VP16 line, where the strong transcriptional activation domain of the herpes virus protein VP16 was fused to the FUL protein. The FUL:VP16 chimeric protein should cause the upregulation of FUL direct targets, overcoming other regulatory effects, as for example the possible repression exerted by other factors or the effect of other FUL interacting proteins in its transcriptional output (Bemer et al., 2017; Balanza et al., 2018). Interestingly, the FUL::FUL:VP16 line ends flowering very early, with the formation of a terminal floral structure (Balanza et al., 2018). *In situ* RNA hybridization indicates that *AG* was expressed ectopically in the periphery of the SAM of the FUL::FUL:VP16 line, just before the formation of the terminal flower (**Figure 6**). Thus, FUL:VP16 was able to bypass the negative regulation exerted by *AP2-like* genes at early inflorescence development, suggesting that FUL could activate directly *AG* expression. In agreement with this observation, when we searched the results of a FUL chromatin immunoprecipitation sequencing data set available in a public repository (NCBI-GEO-DataSet GSE108455, provided by van Mourik, Muiño, Smaczniak, Bemer, Chen, Angenent, and Kaufmann) we found that FUL was able to bind to the *AG* genomic region on two different regions, one centered at approximately 1.5 kb upstream the START codon of the *AG* coding sequence, and another in the distal portion of the second regulatory intron (**Supplementary Figure 2**), which has been described to contain key regulatory elements controlling *AG* (Sieburth and Meyerowitz 1997; Deyholos and Sieburth, 2000; Hong et al., 2003).

## Discussion

**Figure 7:**
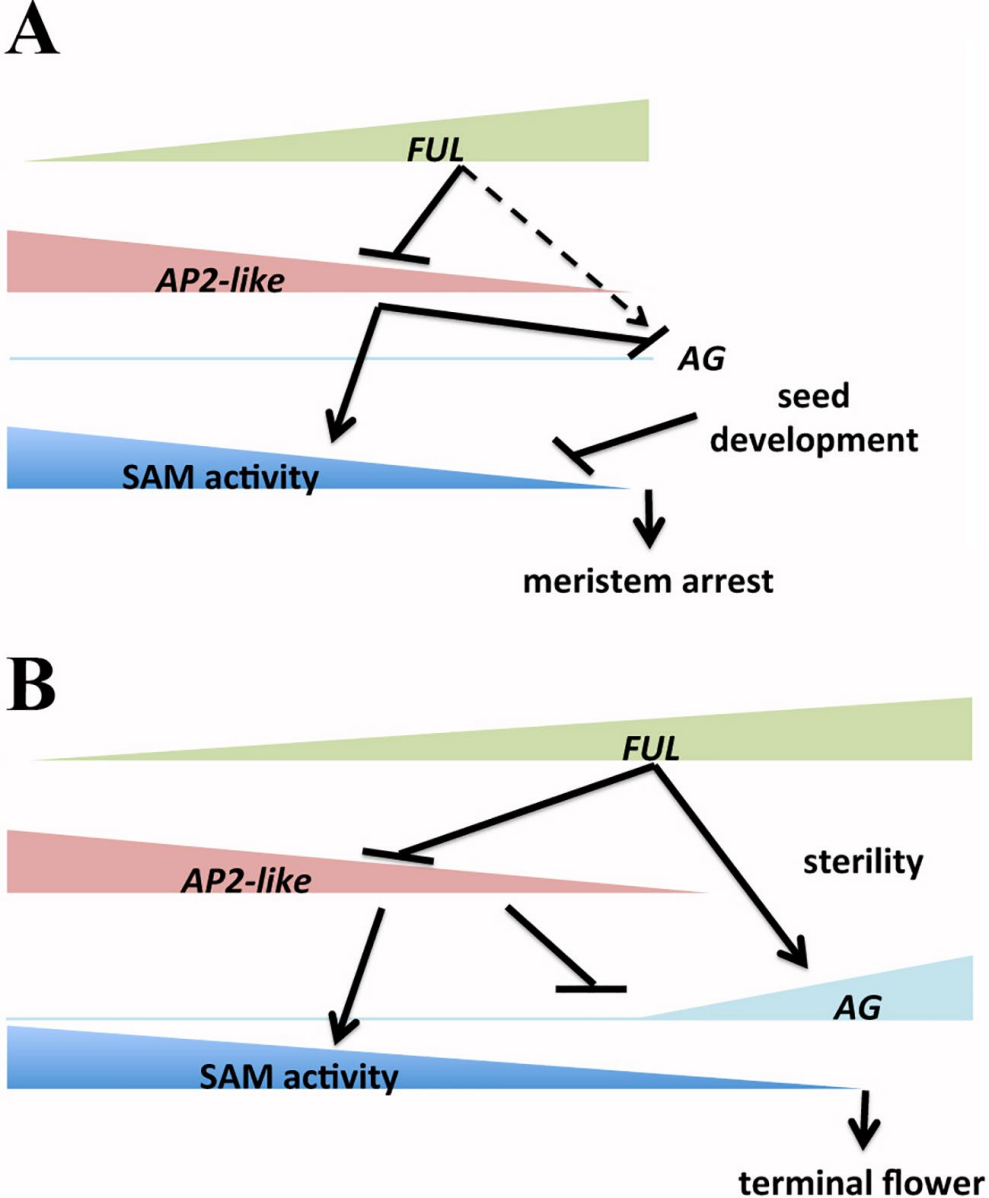
Proposed model for the control of the end of the flowering phase in *Arabidopsis*. **(A)** In normal growth conditions the activity of the inflorescence shoot apical meristem (SAM) is controlled by the FUL-AP2 pathway and the correlative control exerted by the developing seeds. The combined action of both mechanisms induces the meristem arrest and the end of the flowering phase. During inflorescence progression, AP2 and AP2-like proteins control positively SAM meristem activity, and at the same time, repress *AG* expression as well as avoid *AG* activation by FUL in the SAM. **(B)** When the arrest-inductive seed effect is absent (as in sterile mutants or in pruned plants) the inflorescence meristem activity is extended in time. In these conditions, the increasing activity of FUL in the inflorescence meristem should reduce the AP2 and AP2-like levels. The decreasing levels of AP2 proteins would facilitate the direct activation of *AG* by FUL in the SAM, allowing the AG accumulation and the formation of the terminal flower.

The regulated arrest of the flowering phase is still a not well-understood developmental process. In plants with indeterminate inflorescences, seed development is a major factor directing the end of flowering, exerting a negative control over SAM activity. Thus, in the absence of seeds, the length of the inflorescence phase is increased until, at least in some species like *Arabidopsis*, the inflorescence meristem fate changes from indeterminate to determinate (Hensel et al., 1994). In *Arabidopsis*, a genetic mechanism has been also described to control SAM activity and the length of the flowering period in parallel with the correlative control mediated by seeds: the FUL-AP2 pathway. Our results indicate that *FUL* and *AP2-like* genes are also key regulators of the inflorescence meristem fate: FUL appears to induce *AG* activation in the SAM both directly and indirectly, through negative regulation of the *AG* repressors *AP2* and *AP2-like* genes in the SAM. Our results can be easily integrated into the previously proposed model controlling the proliferative arrest of the inflorescence meristems and the end of the flowering phase in *Arabidopsis* (Balanza et al., 2018) (**Figure 7**). When the arrest-inductive seed effect is absent (as in sterile mutants or by pruning of flowers as they are produced), the inflorescence meristem activity is extended in time, forcing the SAM to produce extra flowers. In this scenario, increasing activity of FUL in the inflorescence meristem should simultaneously cause a direct activation of *AG* and a further reduction of the AP2 repressive activity on *AG* in the SAM, which eventually would allow *AG* expression in the inflorescence meristem and the formation of the terminal flower. This model is in agreement with the observed phenotypes of related mutant backgrounds. Thus, the downregulation of *AP2* and *AP2-like* genes in 35S::miR172 lines would cause the early differentiation of the terminal structure, even in the presence of seeds, likely caused by the derepression of *AG* in the SAM. Likewise, in the FUL::FUL:VP16 line, despite the high levels of AP2 (Balanza et al., 2018), the strong upregulation of *AG* in the SAM caused by FUL:VP16 activity would explain the determination of the meristem and the production of a terminal structure consisting of stamens and carpels.

Remarkably, FUL appears to be essential to promote inflorescence meristem determination, since no terminal flowers were formed in *ful* 35S::miR172 mutants or when the *ful* mutation was introduced in lines of very reduced fertility. Together with the likely direct effect on *AG* activation reported in this study, FUL has also been shown to directly upregulate the floral meristem identity gene *LFY* (Balanza et al., 2014). Interestingly, the interplay between LFY, WUS, and AG largely control the determination of the floral meristems. The combined action of WUS and LFY in young floral meristem directly induce *AG* expression in the center of flower buds, where stamens and carpels would differentiate. Once activated, AG represses *WUS* to allow floral meristem termination and the development of the proper set of floral organs in the central whorls of the flower. Accordingly, loss of AG function leads to indeterminate flowers, due to the extended *WUS* activity in the floral meristem (Lenhard et al., 2001; Lohmann et al., 2001).

Because *FUL* is strongly upregulated in the inflorescence meristem at the time of floral transition (Mandel and Yanofsky, 1995a; Hempel et al., 1997; Torti et al., 2012), *LFY* and *AG* activation should be avoided in the SAM to allow the indeterminate growth of the inflorescence and the maintenance of *WUS* expression in the SAM. Our results indicate that terminal flower formation is prevented by *AP2* and *AP2-like* genes, suggesting that these factors could block the FUL-mediated *AG* activation. Thus, the balance between FUL activity (promoting SAM differentiation) and AP2-like activity (preventing SAM differentiation) could be important to maintain the indeterminate fate of the inflorescence. The formation of the terminal flower is premature in the single *ap2* mutant, but it appears even earlier when the expression of all *AP2-like* genes is reduced (35S::miR172 line) suggesting an additive role of the members of this gene family in *AG* repression. Interestingly, the 35S::miR172 plants did not produce a terminal flower immediately after floral transition, despite *FUL* strong upregulation at this time in the SAM (Hempel et al., 1997). This suggests that perhaps FUL levels should be high enough to overcome the AP2 repressive effect, or that additional factors are also acting to prevent inflorescence meristem determination or required to promote floral identity. A clear candidate for this putative function could be TFL1, a repressor of *LFY* in the inflorescence meristem and whose mutation causes the early differentiation of the inflorescence meristem into a flower (Bradley et al., 1997; Ohshima et al., 1997; Ratcliffe et al., 1998). While the functional relationship of FUL and TFL1 has not been precisely elucidated, it has been described that FUL acts by balancing the activities of LFY and TFL1 in the inflorescence (Ferrandiz et al., 2000a). The possible interaction of TFL1 and the FUL-AP2 genetic module in the control of the inflorescence meristem fate, and also on the regulation of the inflorescence proliferative arrest and the length of the flowering phase is of high interest, but remains to be addressed in further studies.

Both *FUL* and *AP2*-*like* genes have been described to be age-regulated by the balance between the miR156 and miR172, with important roles directing developmental phase transitions in plants (Wang et al., 2009; Wu et al., 2009). Phase transitions occur in a gradual and progressive way during early stages of plant development (Wang et al., 2009; Wu et al., 2009). Interestingly, a similar model was also proposed to control inflorescence progression in the past (Schultz and Haughn, 1993; Bradley et al., 1997; Ratcliffe et al., 1998). The moment of the floral transition marks the transition from the vegetative phase to the reproductive phase, and the beginning of inflorescence development. Schultz and Haughn (1993) proposed that during inflorescence development there is a progression of different developmental phases. They also proposed that these inflorescence developmental phases should be controlled by unknown factors that gradually would control the levels of the floral identity genes (Bowman et al., 1993; Schultz and Haughn, 1993). Following this model, the authors distinguished three phases during inflorescence development in *Arabidopsis*: an initial phase where the SAM produces leaves that sustain new branches; a second phase where the SAM produces directly flowers, and a last phase or terminal phase where the SAM differentiates into a flower. It was also discussed that the last phase, the formation of the terminal flower, usually was not observed due to the meristem arrest associated with the end of flowering (Schultz and Haughn, 1993; Bradley et al., 1997; Ratcliffe et al., 1998). Our proposed model is in agreement with the original model proposed by Schultz and Haughn, where the FUL-AP2 pathway could act as the unknown factors that gradually regulate the activation of the floral identity genes in the apical part of the inflorescence. In agreement with the model, *ful* mutant shows an extension on the length of all these phases: it shows a delay in floral transition, a longer first phase of inflorescence development producing extra caulinar leaves (Balanza et al., 2014), an extended second phase with the production of more flowers before meristem arrest (Balanza et al., 2018) and, moreover, it does not enter the last phase (the inflorescence determination) even in the absence of seed production. On the other hand, the *ap2-like* sextuple mutant is early flowering, shortens both phases of inflorescence development (branches and flowers) (Yant et al., 2010; Balanza et al., 2018), and finally enters the last phase developing a terminal flower.

Based in phylogenetic analysis, it has been suggested that determinateness would be the ancestral condition in plants and that indeterminateness could have evolved different times in many species (Stebbins, 1974). Other authors, however, have proposed that indeterminateness could have been acquired very early in evolution and that could have been lost later in some species (Coen and Nugent 1994; Bradley et al., 1997). Our analysis and previous work from other groups indicate that determinateness is facultative in *Arabidopsis* (Chaudhury, 1993; Schultz and Haughn, 1993; Modrusan et al., 1994; Hensel et al., 1994), but hidden by the SAM arrest induced by the correlative control exerted by seeds. From an adaptive point of view, increasing the number of flowers/seeds could provide a clear advantage and increase the reproductive success. On the other hand the production of too many flowers/seeds could also be deleterious, due to the investment of valuable resources to the production of unnecessary seeds, as well as to the increased competition of individuals following seed germination that could affect offspring fitness. According to our observations, *Arabidopsis* could have adopted the strategy to delay the SAM determinacy increasing seed production but at the same time developing a mechanism to limit their final number. Once an optimal number of seeds is produced, a signal could be sensed at the SAM to induce its arrest. This mechanism could also provide an extra benefit. The meristem arrest observed in *Arabidopsis* is reversible (Hensel et al., 1994; Wuest et al., 2016), and if some seed losses are produced, the SAM is able to reactivate and produce new flowers and seeds to ensure further seed production.

In many crops, the end of the flowering phase determines the maximum yield that the plant is able to produce. Thus, understanding the mechanisms that regulate this developmental process could allow the engineering of agronomically important crops in order to increase their yields. Our results complement the scarce information available about the control of the reproductive phase length, as well as provide evidence about how *Arabidopsis* could have evolved an indeterminate inflorescence.

## Materials and Methods

### Plant Material and Growth Conditions

*Arabidopsis thaliana* plants were grown in cabinets at 21°C under LD (16 h light) conditions, illuminated by cool-white fluorescent lamps (150 µE m–2 s–1), in a 1:1:1 by vol. mixture of sphagnum:perlite:vermiculite. To promote germination, seeds were stratified on soil at 4°C for 3 days in the dark. Mutant alleles and transgenic lines have been previously described: *ful-1* (Gu et al., 1998; Balanza et al., 2018), *ful-2* (Ferrandiz et al., 2000a; Balanza et al., 2018), *ap2–12* (Yant et al., 2010) *crc-1* (Bowman and Smyth, 1999), *spt-2* (Alvarez and Smyth, 1999), *pi-1* (Hill and Lord, 1989), quadruple *nga* (Trigueros et al., 2009), 35S::miR172 (Yant et al., 2010), and AG::GUS (Sieburth and Meyerowitz, 1997).

### Induced Sterility/Pruning

After bolting, all flowers produced by the SAM were removed manually every 2–3 days, leaving only the flowers before the anthesis stage. In addition, all the new branches developed by the plant were also removed as they appeared.

### B-Glucuronidase Staining

For GUS histochemical detection, samples were treated for 15 min in 90% ice-cold acetone and then washed for 5 min with washing buffer (25 mM sodium phosphate, 5 mM ferrocyanide, 5 mM ferricyanide, and 1% Triton X-100) and incubated from 4 to 16 h at 37°C with staining buffer (washing buffer+1 mM X-Gluc). Following staining, plant material was fixed, cleared in chloral hydrate, and mounted to be viewed under bright-field microscopy.

### *In Situ* Hybridization

*In situ* hybridizations were performed as described (Ferrandiz et al., 2000a). For hybridization in sections, tissue was fixed for 2 h in FAE solution, dehydrated, embedded, and sectioned to 8 µm. After dewaxing in histoclear and rehydrating, sections were treated for 20 min in 0.2 M HCl, neutralized for 10 min in 2× SSC and then incubated for 30 min with 1 µg/ml Proteinase K at 37°C. Proteinase action was blocked by treating with 2 mg/ml Gly for 5 min and postfixation in 4% formaldehyde for 10 min. Subsequently, sections were dehydrated through an ethanol series before applying the hybridization solution (100 µg/ml transfer RNA; 6× SSC; 3% SDS; 50% formamide, containing approx. 100 ng/µl of antisense digoxigenin-labeled RNA probe), and left overnight at 52°C. Then, sections were washed twice for 90 min in 2× SSC: formamide (50:50) at 52°C before performing the antibody incubation and color detection.

## Data Availability Statement

All datasets for this study are included in the article/**Supplementary Material**.

## Author Contributions

VB, IM-F, SS, MY and CF designed the experiments and interpreted the results. VB and IM-F performed most of the experiments, together with SS. VB and CF wrote the manuscript.

## Funding

This work was supported by grants BIO2015-64531-R (Spanish MINECO/FEDER, UE), RTI2018-099239-B-I00 (Spanish MCIU/AEI/FEDER, UE) and PROMETEU/2019/004 (Generalitat Valenciana) to CF.

## Conflict of Interest

The authors declare that the research was conducted in the absence of any commercial or financial relationships that could be construed as a potential conflict of interest.

## References

[B1] Alvarez J. Smyth D. R. (1999). CRABS CLAW and SPATULA, two *Arabidopsis* genes that control carpel development in parallel with AGAMOUS. Development 126 (11), 2377–2386. 10225997 10.1242/dev.126.11.2377

[B2] Aukerman M. J. Sakai H. (2003). Regulation of flowering time and floral organ identity by a MicroRNA and its APETALA2-like target genes. Plant Cell 15 (11), 2730–2741. 10.1105/tpc.016238 14555699 PMC280575

[B3] Balanza V. Martinez-Fernandez I. Ferrandiz C. (2014). Sequential action of FRUITFULL as a modulator of the activity of the floral regulators SVP and SOC1. J. Exp. Bot. 65 (4), 1193–1203. 10.1093/jxb/ert482 24465009 PMC3935574

[B4] Balanza V. Martinez-Fernandez I. Sato S. Yanofsky M. F. Kaufmann K. Angenent G. C. (2018). Genetic control of meristem arrest and life span in *Arabidopsis* by a FRUITFULL-APETALA2 pathway. Nat. Commun. 9 (1), 565. 10.1038/s41467-018-03067-5 29422669 PMC5805735

[B5] Bemer M. van Mourik H. Muino J. M. Ferrandiz C. Kaufmann K. Angenent G. C. (2017). FRUITFULL controls SAUR10 expression and regulates *Arabidopsis* growth and architecture. J. Exp. Bot. 68 (13), 3391–3403. 10.1093/jxb/erx184 28586421 PMC5853401

[B6] Benlloch R. Berbel A. Serrano-Mislata A. Madueno F. (2007). Floral initiation and inflorescence architecture: a comparative view. Ann. Bot. 100 (3), 659–676. 10.1093/aob/mcm146 17679690 PMC2533596

[B7] Blazquez M. A. Soowal L. N. Lee I. Weigel D. (1997). LEAFY expression and flower initiation in Arabidopsis. Development 124 (19), 3835–3844. 9367439 10.1242/dev.124.19.3835

[B8] Bowman J. L. Smyth D. R. (1999). CRABS CLAW, a gene that regulates carpel and nectary development in *Arabidopsis*, encodes a novel protein with zinc finger and helix-loop-helix domains. Development 126, 11, 2387–2396. 10.1126/science.275.5296.80 10225998

[B9] Bowman J. L. Drews G. N. Meyerowitz E. M. (1991). Expression of the *Arabidopsis* floral homeotic gene AGAMOUS is restricted to specific cell types late in flower development. Plant Cell 3 (8), 749–758. 10.1105/tpc.3.8.749 1726485 PMC160042

[B10] Bowman J. L. Alvarez J. Weigel D. Meyerowitz E. M. Smyth D. R. (1993). Control of flower development in *Arabidopsis thaliana* by APETALA1 and interacting genes. Development 119 (3), 721. 10.1105/tpc.3.8.749

[B11] Bradley D. Ratcliffe O. Vincent C. Carpenter R. Coen E. (1997). Inflorescence commitment and architecture in *Arabidopsis*. Science 275 (5296), 80–83. 10.1105/tpc.5.101277 8974397

[B12] Chaudhury A. M. (1993). Nuclear genes controlling male fertility. Plant Cell 5 (10), 1277–1283. 10.1126/science.1088060 12271027 PMC160360

[B13] Chen X. (2004). A microRNA as a translational repressor of APETALA2 in *Arabidopsis* flower development. Science 303 (5666), 2022–2025. 10.1105/tpc.12.101799 12893888 PMC5127708

[B14] Coen E. S. Nugent J. M. (1994). Evolution of flowers and inflorescences. Development 1994, 107. 10.1016/0092-8674(91)90551-9

[B15] Deyholos M. K. Sieburth L. E. (2000). Separable whorl-specific expression and negative regulation by enhancer elements within the AGAMOUS second intron. Plant Cell 12 (10), 1799–1810. 10.1105/tpc.12.10.1799 11041877 PMC149120

[B16] Drews G. N. Bowman J. L. Meyerowitz E. M. (1991). Negative regulation of the *Arabidopsis* homeotic gene AGAMOUS by the APETALA2 product. Cell 65 (6), 991–1002. 10.1126/science.289.5478.436 1675158

[B17] Ferrandiz C. Gu Q. Martienssen R. Yanofsky M. F. (2000a). Redundant regulation of meristem identity and plant architecture by FRUITFULL, APETALA1 and CAULIFLOWER. Development 127 (4), 725–734. 10648231 10.1242/dev.127.4.725

[B18] Ferrandiz C. Liljegren S. J. Yanofsky M. F. (2000b). Negative regulation of the SHATTERPROOF genes by FRUITFULL during *Arabidopsis* fruit development. Science 289 (5478), 436–438. 10.1126/science.289.5478.436 10903201

[B19] Gu Q. Ferrandiz C. Yanofsky M. F. Martienssen R. (1998). The FRUITFULL MADS-box gene mediates cell differentiation during *Arabidopsis* fruit development. Development 125 (8), 1509–1517. 10.1104/pp.106.3.863 9502732

[B20] Hempel F. D. Weigel D. Mandel M. A. Ditta G. Zambryski P. C. Feldman L. J. (1997). Floral determination and expression of floral regulatory genes in *Arabidopsis*. Development 124 (19), 3845–3853. 10.1139/b89-375 9367440

[B21] Hensel L. L. Nelson M. A. Richmond T. A. Bleecker A. B. (1994). The fate of inflorescence meristems is controlled by developing fruits in *Arabidopsis*. Plant Physiol. 106 (3), 863–876. 10.1105/tpc.009548 7824655 PMC159609

[B22] Hill J. P. Lord E. M. (1989). Floral development in *Arabidopsis thaliana*: a comparison of the wild type and the homeotic pistillata mutant. Can. J. Bot. 67 (10), 2922–2936. 10.1139/b89-375

[B23] Hong R. L. Hamaguchi L. Busch M. A. Weigel D. (2003). Regulatory elements of the floral homeotic gene AGAMOUS identified by phylogenetic footprinting and shadowing. Plant Cell 15 (6), 1296–1309. 10.1105/tpc.009548 12782724 PMC156367

[B24] Imamura T. Nakatsuka T. Higuchi A. Nishihara M. Takahashi H. (2011). The gentian orthologs of the FT/TFL1 gene family control floral initiation in Gentiana. Plant Cell Physiol. 52 (6), 1031–1041. 10.1104/pp.86.3.978 21531759

[B25] Iwata H. Gaston A. Remay A. Thouroude T. Jeauffre J. Kawamura K. (2012). The TFL1 homologue KSN is a regulator of continuous flowering in rose and strawberry. Plant J. 69 (1), 116–125. 10.1111/j.1365-313X.2011.04776.x 21895811

[B26] Kelly M. O. Davies P. J. (1988). Photoperiodic and genetic control of carbon partitioning in peas and its relationship to apical senescence. Plant Physiol. 86 (3), 978–982. 10.1104/pp.86.3.978 16666020 PMC1054606

[B27] Laux T. Mayer K. F. Berger J. Jurgens G. (1996). The WUSCHEL gene is required for shoot and floral meristem integrity in *Arabidopsis*. Development 122 (1), 87–96. 10.1104/pp.34.5.570 8565856

[B28] Lenhard M. Bohnert A. Jurgens G. Laux T. (2001). Termination of stem cell maintenance in *Arabidopsis* floral meristems by interactions between WUSCHEL and AGAMOUS. Cell 105 (6), 805–814. 10.1105/tpc.11.61007 11440722

[B29] Leopold A. C. Niedergang-Kamien E. Janick J. (1959). Experimental Modification of Plant Senescence. Plant Physiol. 34 (5), 570–573. 10.1104/pp.59.61136 16655275 PMC541254

[B30] Liljegren S. J. Gustafson-Brown C. Pinyopich A. Ditta G. S. Yanofsky M. F. (1999). Interactions among APETALA1, LEAFY, and TERMINAL FLOWER1 specify meristem fate. Plant Cell 11 (6), 1007–1018. 10.1016/S0092-8674(01)00384-1 10368173 PMC144247

[B31] Lindoo S. J. Nooden L. D. (1977). Studies on the behavior of the senescence signal in anoka soybeans. Plant Physiol. 59 (6), 1136–1140. 10.1038/360273a0 16660009 PMC542522

[B32] Lohmann J. U. Hong R. L. Hobe M. Busch M. A. Parcy F. Simon R. (2001). A molecular link between stem cell regulation and floral patterning in *Arabidopsis*. Cell 105 (6), 793–803. 10.1016/s0092-8674(01)00384-1 11440721

[B33] Mandel M. A. Yanofsky M. F. (1995a). The Arabidopsis AGL8 MADS box gene is expressed in inflorescence meristems and is negatively regulated by APETALA1. Plant Cell 7 (11), 1763–1771. 10.1016/S0092-8674(00)81703-1 8535133 PMC161036

[B34] Mandel M. A. Yanofsky M. F. (1995b). A gene triggering flower formation in *Arabidopsis*. Nature 377 (6549), 522–524. 10.1007/s00299-011-1057-3 7566148

[B35] Mandel M. A. Gustafson-Brown C. Savidge B. Yanofsky M. F. (1992). Molecular characterization of the *Arabidopsis* floral homeotic gene APETALA1. Nature 360 (6401), 273–277. 10.1038/377522a0 1359429

[B36] Mayer K. F. Schoof H. Haecker A. Lenhard M. Jurgens G. Laux T. (1998). Role of WUSCHEL in regulating stem cell fate in the *Arabidopsis* shoot meristem. Cell 95 (6), 805–815. 10.1016/0092-8674(92)90271-D 9865698

[B37] Mimida N. Ureshino A. Tanaka N. Shigeta N. Sato N. Moriya-Tanaka Y. (2011). Expression patterns of several floral genes during flower initiation in the apical buds of apple (Malus x domestica Borkh.) revealed by in situ hybridization. Plant Cell Rep. 30 (8), 1485–1492. 10.1105/tpc.6.3.333 21424812

[B38] Mizukami Y. Ma H. (1992). Ectopic expression of the floral homeotic gene AGAMOUS in transgenic *Arabidopsis* plants alters floral organ identity. Cell 71 (1), 119–131. 10.1111/j.1365-313X.2010.04185.x 1356630

[B39] Modrusan Z. Reiser L. Feldmann K. A. Fischer R. L. Haughn G. W. (1994). Homeotic Transformation of Ovules into Carpel-like Structures in *Arabidopsis*. Plant Cell 6 (3), 333–349. 10.1104/pp.1.1.3 12244239 PMC160437

[B40] Mohamed R. Wang C. T. Ma C. Shevchenko O. Dye S. J. Puzey J. R. (2010). Populus CEN/TFL1 regulates first onset of flowering, axillary meristem identity and dormancy release in Populus. Plant J. 62 (4), 674–688. 10.1016/B978-012520915-1/50018-7 20202169

[B41] Murneek A. E. (1926). Effects of Correlation between Vegetative and Reproductive Functions in the Tomato (Lycopersicon Esculentum Mill.). Plant Physiol. 1 (1), 3–56 57. 10.1007/s004380050407 16652464 PMC441333

[B42] Noodén L. D. Guiamét J. J. John I. (2004). “15 - Whole Plant Senescence,” in Plant Cell Death Processes. Ed. Noodén L. D. (San Diego: Academic Press), 227–244. 10.1016/B978-012520915-1/50018-7

[B43] Ohshima S. Murata M. Sakamoto W. Ogura Y. Motoyoshi F. (1997). Cloning and molecular analysis of the *Arabidopsis* gene Terminal Flower 1. Mol. Gen. Genet. 254 (2), 186–194. 10.1007/s004380050407 9108281

[B44] Pnueli L. Carmel-Goren L. Hareven D. Gutfinger T. Alvarez J. Ganal M. (1998). The SELF-PRUNING gene of tomato regulates vegetative to reproductive switching of sympodial meristems and is the ortholog of CEN and TFL1. Development 125 (11), 1979–1989. 9570763 10.1242/dev.125.11.1979

[B45] Ratcliffe O. J. Amaya I. Vincent C. A. Rothstein S. Carpenter R. Coen E. S. (1998). A common mechanism controls the life cycle and architecture of plants. Development 125 (9), 1609–1615. 10.1038/nbt1754 9521899

[B46] Ratcliffe O. J. Bradley D. J. Coen E. S. (1999). Separation of shoot and floral identity in *Arabidopsis*. Development 126 (6), 1109–1120. 10021331 10.1242/dev.126.6.1109

[B47] Robinson J. T. Thorvaldsdottir H. Winckler W. Guttman M. Lander E. S. Getz G. (2011). Integrative genomics viewer. Nat. Biotechnol. 29 (1), 24–26. 10.1105/tpc.3.9.877 21221095 PMC3346182

[B48] Schultz E. A. Haughn G. W. (1993). Genetic analysis of the floral initiation process (FLIP) in *Arabidopsis*. Development 119 (3), 745–765. 10.1093/pcp/pcp148

[B49] Shannon S. Meeks-Wagner D. R. (1991). A Mutation in the *Arabidopsis* TFL1 gene affects inflorescence meristem development. Plant Cell 3 (9), 877–892. 10.1105/tpc.9.3.355 12324621 PMC160057

[B50] Shikata M. Koyama T. Mitsuda N. Ohme-Takagi M. (2009). *Arabidopsis* SBP-box genes SPL10, SPL11 and SPL2 control morphological change in association with shoot maturation in the reproductive phase. Plant Cell Physiol. 50 (12), 2133–2145. 10.1126/science.189.4202.565 19880401

[B51] Sieburth L. E. Meyerowitz E. M. (1997). Molecular dissection of the AGAMOUS control region shows that cis elements for spatial regulation are located intragenically. Plant Cell 9 (3), 355–365. 10.1105/tpc.111.092791 9090880 PMC156923

[B52] Sinclair T. R. de Wit C. T. (1975). Photosynthate and nitrogen requirements for seed production by various crops. Science 189 (4202), 565–567. 10.1105/tpc.109.065508 17798304

[B53] Stebbins G. L. (1974). Flowering plants : evolution above the species level (Cambridge, Mass: Belknap Press of Harvard University Press). 10.1002/9781119312994.apr0640

[B54] Torti S. Fornara F. Vincent C. Andres F. Nordstrom K. Gobel U. (2012). Analysis of the *Arabidopsis* shoot meristem transcriptome during floral transition identifies distinct regulatory patterns and a leucine-rich repeat protein that promotes flowering. Plant Cell 24 (2), 444–462. 10.1016/j.cell.2009.06.014 22319055 PMC3315226

[B55] Trigueros M. Navarrete-Gomez M. Sato S. Christensen S. K. Pelaz S. Weigel D. (2009). The NGATHA genes direct style development in the *Arabidopsis* gynoecium. Plant Cell 21 (5), 1394–1409. 10.1016/0092-8674(92)90295-N 19435937 PMC2700528

[B56] Walker C. H. Bennett T. (2018). “Forbidden Fruit: Dominance Relationships and the Control of Shoot Architecture,” in Annual Plant Reviews online. Ed. Roberts J. A.,(Oxford, UK: John Wiley and Sons, Ltd) 1–38. 10.1126/science.261.51291723

[B57] Wang J. W. Czech B. Weigel D. (2009). miR156-regulated SPL transcription factors define an endogenous flowering pathway in *Arabidopsis thaliana*. Cell 138 (4), 738–749. 10.1126/science.1114358 19703399

[B58] Weigel D. Meyerowitz E. M. (1993). Activation of floral homeotic genes in *Arabidopsis*. Science 261 (5129), 1723–1726. 10.1016/j.cell.2009.06.031 17794879

[B59] Weigel D. Alvarez J. Smyth D. R. Yanofsky M. F. Meyerowitz E. M. (1992). LEAFY controls floral meristem identity in *Arabidopsis*. Cell 69 (5), 843–859. 10.1111/j.1399-3054.1997.tb00567.x 1350515

[B60] Wigge P. A. Kim M. C. Jaeger K. E. Busch W. Schmid M. Lohmann J. U. (2005). Integration of spatial and temporal information during floral induction in *Arabidopsis*. Science 309 (5737), 1056–1059. 10.1104/pp.15.01995 16099980

[B61] Wilson J. B. (1997). An evolutionary perspective on the ‘death hormone’hypothesis in plants. Physiologia Plantarum 99 (3), 511–516. 10.1016/j.devcel.2009.06.007

[B62] Wu G. Park M. Y. Conway S. R. Wang J. W. Weigel D. Poethig R. S. (2009). The sequential action of miR156 and miR172 regulates developmental timing in *Arabidopsis*. Cell 138 (4), 750–759. 10.1038/346035a0 19703400 PMC2732587

[B63] Wuest S. E. Philipp M. A. Guthorl D. Schmid B. Grossniklaus U. (2016). Seed Production Affects Maternal Growth and Senescence in *Arabidopsis*. Plant Physiol. 171 (1), 392–404. 10.1105/tpc.110.075606 27009281 PMC4854700

[B64] Yamaguchi A. Wu M. F. Yang L. Wu G. Poethig R. S. Wagner D. (2009). The microRNA-regulated SBP-Box transcription factor SPL3 is a direct upstream activator of LEAFY, FRUITFULL, and APETALA1. Dev. Cell 17 (2), 268–278. 10.1016/j.devcel.2009.06.007 19686687 PMC2908246

[B65] Yanofsky M. F. Ma H. Bowman J. L. Drews G. N. Feldmann K. A. Meyerowitz E. M. (1990). The protein encoded by the *Arabidopsis* homeotic gene agamous resembles transcription factors. Nature 346 (6279), 35–39. 10.1038/346035a0 1973265

[B66] Yant L. Mathieu J. Dinh T. T. Ott F. Lanz C. Wollmann H. (2010). Orchestration of the floral transition and floral development in *Arabidopsis* by the bifunctional transcription factor APETALA2. Plant Cell 22 (7), 2156–2170. 10.1105/tpc.110.075606 20675573 PMC2929098

