# Supplementary material for: Inflorescence Meristem Fate Is Dependent on Seed Development and FRUITFULL in Arabidopsis thaliana
Source: Front Plant Sci. 2019 Dec 18;10:1622. doi: 10.3389/fpls.2019.01622 (PMC6930240; doi:10.3389/fpls.2019.01622)
Supplement: Supplementary file 1 [file DataSheet_1.docx]

**Supplementary material**


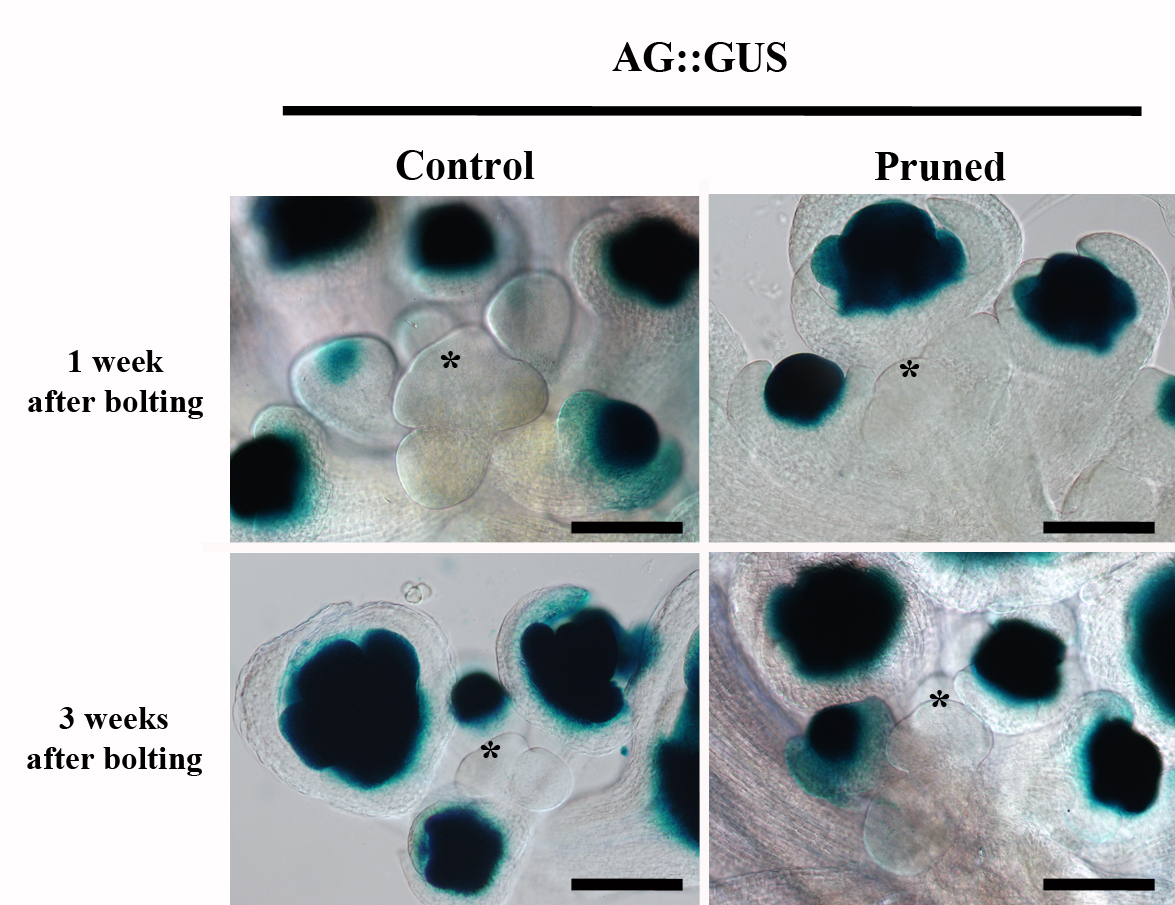


**Supplementary Figure 1: AG::GUS reporter activity in proliferative inflorescence meristems of wildtype plants**. At early stages of inflorescence development, when flower production is actively sustained, AG::GUS is never detected in the SAM, regardless of seed production. The staining pattern is very similar in untreated plants (control) and in those where flowers were removed as they were formed (pruned).


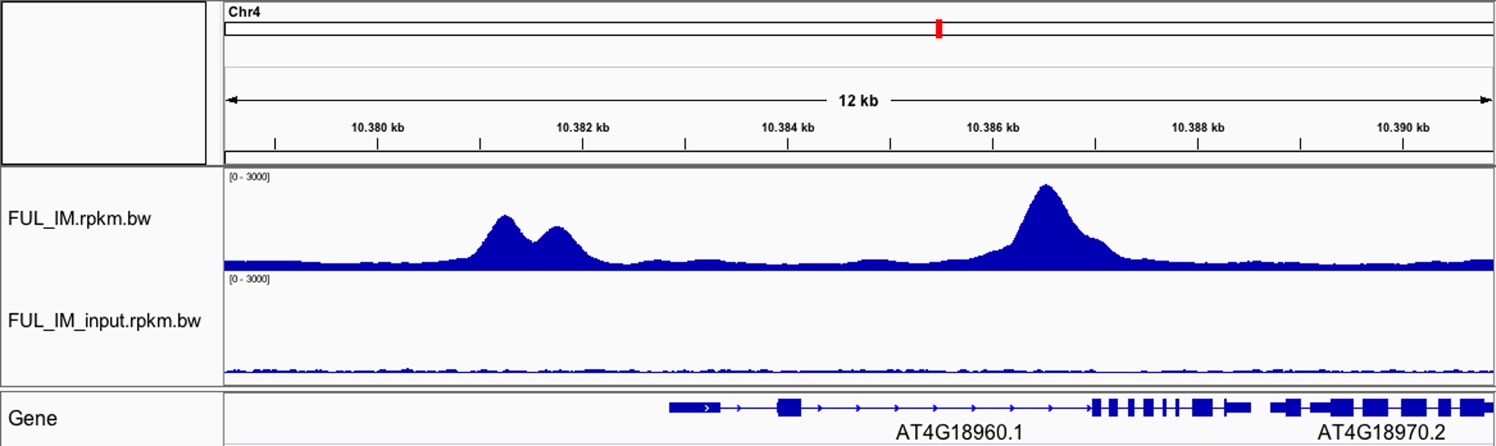


**Supplementary Figure 2: Binding sites observed for FUL on the AG genomic region**. Data from the NCBI-GEO-DataSet GSE108455 were analyzed with the IGV application (Robinson et al. 2011), identifying two regions bound by FUL, first 1.5 kb upstream the AG ATG, and a second region at the end of the second regulatory intron of AG.
